# Barriers to the use of direct access according to allied health professionals; an exploration among Dutch physiotherapists, dietitians, and health insurers

**DOI:** 10.1186/s12875-025-02816-y

**Published:** 2025-04-25

**Authors:** Laura J. Damen, Britt Beerman, W. M. Meijer, B. J. Knottnerus, J. D. De Jong, L. H. D. Van Tuyl

**Affiliations:** 1 Nivel, Netherlands Institute for Health Services Research, Utrecht, the Netherlands; 2 CAPHRI, Maastricht University, PO Box 616, 6200 MD Maastricht, the Netherlands

**Keywords:** Primary care, Allied health professionals, Direct access, Healthcare system, Qualitative research

## Abstract

**Introduction:**

General practices experience high workloads and increasing volume of patients. But not all patients require GP care. We previously reported that GPs observed that allied health professionals frequently request referrals even when patients initially seek direct access to care. This study, therefore, explored the barriers to the use of direct access as identified by allied health professionals and health insurers.

**Methods:**

Seventeen in-depth interviews were conducted comprising six dietitians, seven physiotherapists, and four health insurers. The interviews were recorded, transcribed, and analysed using the qualitative research principles of thematic analysis.

**Results:**

The main key themes that derived from the interviews included: (1) policy, (2) motivation, and (3) public profile, which were further subdivided into sub-themes. While health insurers claimed to not impose any specific requirements for the use of direct access, allied health professionals faced several policy-related challenges with direct access. For instance, dietitians reported reduced treatment time under direct access and claimed lower reimbursement for intake appointments compared to those following referrals—an assertion denied by insurers. Other reasons for not using direct access include greater uncertainty about potentially overlooking health issues during the initial intake. Additionally, some physiotherapists and dietitians perceive direct access as less convenient than obtaining a referral, for example as it involves fewer regulations from health insurers and therefore saves time. Public profile also played a role in the use of direct access. Dietitians noted limited patient awareness of their services and the availability of direct access, unlike physiotherapists, who benefited from public campaigns and effective management strategies.

**Conclusions:**

Despite the small sample size, this study showed a gap between the perspectives of allied health professionals and the health insurers that need to be further explored. Direct access has inadvertently increased the workload for some allied health professionals, particularly dietitians. This is an unintended outcome of a system intended, ironically, to reduce pressure on care. The use of direct access could be improved by carrying out further investigations and addressing the challenges faced by allied health professionals.

**Supplementary Information:**

The online version contains supplementary material available at 10.1186/s12875-025-02816-y.

## Introduction

Healthcare systems around the world are facing pressure due to the increasing demand for, and costs of, healthcare [1–4]. At the same time, there is a shortage of workforce [4–6]. This has resulted in mounting pressure on primary care. This has manifested itself in a reduced quality of care, longer waiting times, and elevated burnout rates among primary care providers [7, 8]. General Practitioner (GP) care, in particular, is affected [8]. One approach to organise care more efficiently and relieve pressure on GP care is to expand direct accessibility to allied health professionals [9–11]. Such direct access is already available in various countries such as the Netherlands, the United Kingdom, the United States, and Denmark [10]. It allows patients to seek care from allied health professionals, such as physiotherapists, without needing a referral from a GP or another healthcare professional (HCP). During an initial appointment without a referral, the allied health professional evaluates whether treatment is appropriate and safe. They do a screening for any, so called, red flags, which are symptoms or conditions outside the scope of their practice. In such cases, the allied health professional refers the patient to their GP. Previous research in musculoskeletal physiotherapy suggests that patients who access care directly often receive a comparable or even higher quality of care in terms of patient-reported satisfaction, condition improvement, and timely referral to another healthcare professional, compared to those whose initial contact is with a GP or another HCP [9, 12–15]. Additionally, many patients recognise the time saved by bypassing the GP and directly consulting an allied health professional as an advantage [16].

However, in a recent interview study we conducted, Dutch GPs reported that referrals to allied health professionals remain common [11]. Patients visit their GP for referrals even when they could have accessed allied health services directly, often due to a lack of awareness about direct access or uncertainty about which healthcare professional to consult [16]. Additionally, GPs observed that allied health professionals often request formal referrals for patients who initially sought care via direct access. The reasons for these requests, however, are unclear to the GP [11]. An earlier Dutch study by an agency advising on healthcare organisation explored the costs associated with primary care and briefly touched upon the perceptions of allied health professionals regarding restrictions on direct access [17]. During interviews, some allied health professionals expressed a reluctance to use direct access due to limited or absent reimbursement for screening, as well as concerns about the reduction in the time available for treatment due to screening obligations. These findings suggest that the reasons for requesting referrals may be influenced by the policies of health insurers. Health insurers may also impose restrictions on direct access, potentially contributing to differences between practices in its use [18]. Understanding the barriers allied health professionals face when accepting patients using direct access is essential for identifying ways to expand its use. Despite its importance, there is a notable lack of in-depth research on this subject. Moreover, including the perspectives of both health insurers and allied health professionals is crucial, as this aspect has been overlooked previously.

This study aimed to explore the barriers to the use of direct access as identified by physiotherapists, dietitians, and health insurers. We focused on these specific allied health professionals due to differences in their use of direct access. In 2023, 72.8% of patients accessed physiotherapy through direct access, while only 13.4% did so for dietetic services [19, 20]. Moreover, we investigated the perspectives of health insurers in order to understand their policies on direct access. By gaining deeper insights into the views of allied health professionals and health insurers, policy decisions can be informed that promote the expansion of direct access. Our research seeks to answer the following question: *What are the barriers to the use of direct access according to dietitians*,* physiotherapists*,* and health insurers?*

## Method

### Setting

This study was performed in the Netherlands where care can be provided for many conditions both by a physiotherapist and a dietitian without a referral from a GP or another doctor. Direct access to physiotherapy has been available since 2006, while other allied health services, such as speech therapy and dietetics, have been directly accessible since 2011 [19, 21, 22]. The insurance coverage however, differs between allied health professionals. For physiotherapy, the majority of the treatments are not included in the basic health insurance package, but it is possible to have an additional insurance that covers a predetermined number of treatments [23]. For dietitian care, three hours of general treatment are covered each year in the basic health insurance package, though reimbursement will only commence after the deductible or own risk costs have been surpassed [24]. Allied health professionals are reimbursed by health insurers on a fee-for-service basis, depending on policy conditions and contractual agreements.

### Design

A descriptive qualitative design was used to explore the barriers to the use of direct access according to dietitians, physiotherapists, and health insurers.

### Sample and recruitment

Under the assumption that allied healthcare professionals have comparable experiences with direct access, we aimed to include a sample of 12 professionals comprising six physiotherapists and six dietitians. Literature suggests that data saturation in qualitative studies using in-depth interviews is often reached within a range of nine to 17 participants [25]. Therefore, we started with this number of participants. Allied health professionals were eligible if they were currently practising in primary care. Additionally, we targeted a sample of four representatives from the health insurers, to which we hereafter refer to as health insurers. In the Netherlands, there are ten health insurance companies, divided into four larger ones, covering 85% of the population. The other 15% are insured by six smaller health insurance companies. We included in our sample two larger and two smaller ones [26]. Representatives of health insurers were eligible if they had expertise in allied healthcare procurement.

The allied healthcare providers were recruited through a mix of convenience and purposive sampling while the health insurers were recruited solely through purposive sampling. Purposive sampling involved reaching out to contacts in Nivel’s (Netherlands Institute for Health Services Research) network. Convenience sampling, meanwhile, included study invitations shared in provider association newsletters and on websites. Those allied healthcare professionals and health insurers who applied first, were approached for the interviews.

During one of the interviews, one participant disclosed that she was not currently practising as a physiotherapist, although she was still employed within a physiotherapy practice and intended to return to her role as a physiotherapist soon. Given the relevance of her insights, we decided not to exclude her from the study. We included her and recruited an additional physiotherapist, resulting in seven physiotherapists instead of the planned six. In total, we interviewed four health insurers, and 13 allied healthcare professionals.

### Data collection

We used semi-structured interview guides (Appendix A, B). The interview guides were developed by the first author (LD) who already has experience performing interviews and is an experienced occupational therapist, with practical experience with direct access to care. Feedback from the research team was incorporated in order to refine the guides. The guides are based on the consolidated framework for advancing implementation research (CFIR), a model designed for the development and validation of implementation theories. CFIR provides a systematic approach to assessing potential barriers and facilitators, helping to identify what works, where, and why across different contexts [27, 28]. In this study the implementation evaluated is the use of direct access for physiotherapists and dietitians. The framework consists of five domains: intervention characteristics, for example the advantages of direct access; outer setting, for example the reasons for patients to choose or not choose direct access; inner setting, for example the policy within practice regarding direct access; the characteristics of individuals involved, for example the preference for direct access or a referral of the allied health professional; and, the process of implementation, for example the promotion of direct access. Questions were added to the topic list for each domain relevant for the study. One pilot interview was performed with a physiotherapist and the feedback was used to adjust the guide.

The interviews with allied healthcare professionals and the health insurers were performed interchangeably. In this way we could discuss information derived from one interview, in the next interview. Thus the interview guide was developed further based on complete interviews, which was an iterative process. For example, the barriers to the use of direct access related to the health insurer mentioned by the allied healthcare professionals, were verified in the interviews with the health insurers. All of the interviews took place in August and September 2024. The interviews were conducted by the first (LD) or second author (BB). For calibration purposes, the first interview was conducted by both authors to ensure a homogeneous approach. Also, the interview with the last health insurer was conducted together to complement each other with information from previous interviews. The interviews were conducted online and had a maximum duration of 45 min. The sessions were recorded on audio. After the interview, a summary was sent to each participant allowing them to provide feedback.

### Analysis

The interviews were distributed between the first and second authors, transcribed verbatim, and anonymised. For the process of the analysis, the data collected was imported in the software program MaxQDA 2024. The transcripts of the interviews were subjected to inductive thematic analysis, involving the following steps: becoming familiar with the data; generating initial codes; searching for themes; reviewing themes; defining and naming themes; and, producing the report [29]. We modified the process by incorporating intercoder reliability as an additional step. This modification was made to enhance the credibility of the findings and to contribute to a broader and more nuanced understanding of the data [30, 31].

Two interviews with the physiotherapists, dietitians, and health insurers were analysed independently by the first and second authors (LD and BB). Discrepancies in coding were discussed until a consensus was achieved, leading to the creation of a concise codebook (Appendix C, D) for subsequent analysis. The remaining transcripts were analysed by the first author (LD). Before introducing a new code, LD discussed this with BB to reach agreement. Additionally, the second author (BB) randomly reviewed six interviews in order to verify the coding. Any discrepancies were resolved through discussion.

After coding the transcripts, LD and BB started developing the themes from the interviews with the allied health professionals. This was carried out in consultation with the research team. A similar process was then applied to the interviews with health insurers, which focused on their policies regarding direct access—a topic also discussed by the allied health professionals. During the interviews with health insurers, efforts were made to clarify the points raised by the allied health professionals. This prompted discussions on the same subjects and the identification of consistent topics across both groups. The themes derived from the health insurers’ interviews were therefore found to align closely with those from the allied health professionals. Quotes included in the results were translated from Dutch.

### The trustworthiness of the study

To assure the trustworthiness of the study four key criteria were taken into account: credibility, transferability, dependability and confirmability [31]. Two authors (LD and BB) independently reviewed six interviews to address their credibility. In addition, researcher triangulation was performed throughout the whole process of analysing the data and writing the results, by discussing the interpretation of the results multiple times with the research team which consisted of members with different backgrounds to ensure the criteria’s credibility. Furthermore, to improve the credibility, a member check was carried out on every participant in order to validate their feedback. This was useful for checking our interpretation of the results. Additionally, a peer debriefing was organised with a group of peer researchers who were not directly involved in the study to review the article. The credibility is also about the fit between the respondents’ views and the researchers’ representation of them. To improve this, we adapted the interview guide during the process of interviewing to make it more suitable to the knowledge we gained during the process.

We enhanced the transferability of the study by providing detailed information in the method section on both the participants and the context. This means that others, who are interested in transferring the findings to their own setting, can judge it. The use of a semi-structured interview guide also contributes to the transferability since it helps other researchers to ask the same questions. The dependability was assured by detailed reporting of the process of the study and using the criteria for reporting qualitative research (COREQ). This is a checklist for reporting important aspects of qualitative studies [30]. By including verbatim statements from the participants in the results section, the degree to which the criteria can be confirmed was improved [31]. This was also enhanced by checking information derived from one interview with the next one.

### Ethical procedures

Approval by a medical ethics committee is not needed for non-experimental interview data involving experts, in this case, allied health professionals and health insurers, according to Dutch law [32]. All the respondents received an informed consent form before the start of the interview and the recording. Furthermore, we checked whether the respondents had read the informed consent form and if they had any questions with regard to the form or the study. Subsequently, they were asked to confirm their agreement with the terms of the informed consent. This confirmation was obtained on the recording. All participants gave verbal informed consent. To secure the privacy of the participants, the data was anonymised. Also, the data was saved on a secured server of the researchers research institute in order to protect the data retrieved from the interviews.

## Results

We conducted 17 in-depth interviews with seven physiotherapists, six dietitians, and four health insurers. The background characteristics of the allied health professionals are shown in Table 1 and those of the health insurers are shown in Table 2. Most of the allied health professionals participating were over 50 years old and had more than ten years of experience. By contrast, most of the health insurers were 39 years or younger with less than six years of experience.

**Table 1:** Background characteristics of allied health professionals in the interviews (*N* = 13)

Allied health professionals	
• Physiotherapists	7^1^
• Dietitians	6
Gender	
• Male	4
• Female	9
Age	
• 39 and younger	3
• 40–49	1
• 50–59	4
• 60 and older	5
Owner of a practice	
• Yes	6
• No	7
Years of experience working as an allied health professional	
• 0–5	2
• 6–10	1
• More than 10	10
How urban the area in which the practice is located is^2^	
• Highly urbanised	8
• Slightly urbanised	1
• Less urban/rural	4

Notes:

1) One participant was not currently practising as a physiotherapist, although she was still employed within a practice and intended to return to the role as a physiotherapist soon.

2) Highly urbanised is an address density of 1,500 addresses or more per km2. Slightly urbanised is an address density of 1,000 to 1,500 addresses per km2. Less urbanised is an address density of a maximum of 1,000 addresses per km2 [33]

**Table 2:** Background characteristics of health insurers in the interviews (*N* = 4)

Size of the health insurance company^1^	
• Small	2
• Large	2
Age	
• 39 and younger	3
• 40–49	1
• 50–59	0
• 60 and older	0
Years of experience working at a health insurance company	
• 0–5	3
• 6–10	1
• More than 10	0

Note: 1) In the Netherlands, there are in total ten health insurance companies, divided into four larger ones insuring 85% of the people and the other 15% are insured at six other health insurance companies [26]

The allied health professionals generally highlighted similar aspects regarding the use of direct access. They identified several benefits, such as saving GPs’ time, providing convenience for patients, reducing waiting times for patients, and potentially saving societal costs by directing patients to the appropriate care more efficiently. However, their perspectives on direct access varied between the professions. Physiotherapists often expressed no strong preference between direct access and referrals. When they did have a preference, it typically leaned toward direct access. They recognised both the advantages and disadvantages of each approach, but overall, they did not report significant issues with either and felt that both systems worked well for their practice. Most dietitians, on the other hand, tended to favour referrals over direct access. They also questioned whether they could match physiotherapists’ direct access rates due to the frequent need for a GP’s preliminary assessment to address underlying health conditions. By contrast, they noted physiotherapy typically involves more direct, externally focused, interventions.

Health insurers reported that they facilitate direct access, emphasising its benefits in alleviating pressure on GPs, reducing healthcare costs by guiding patients directly to appropriate care providers, and enhancing overall efficiency.

**Fig. 1:**
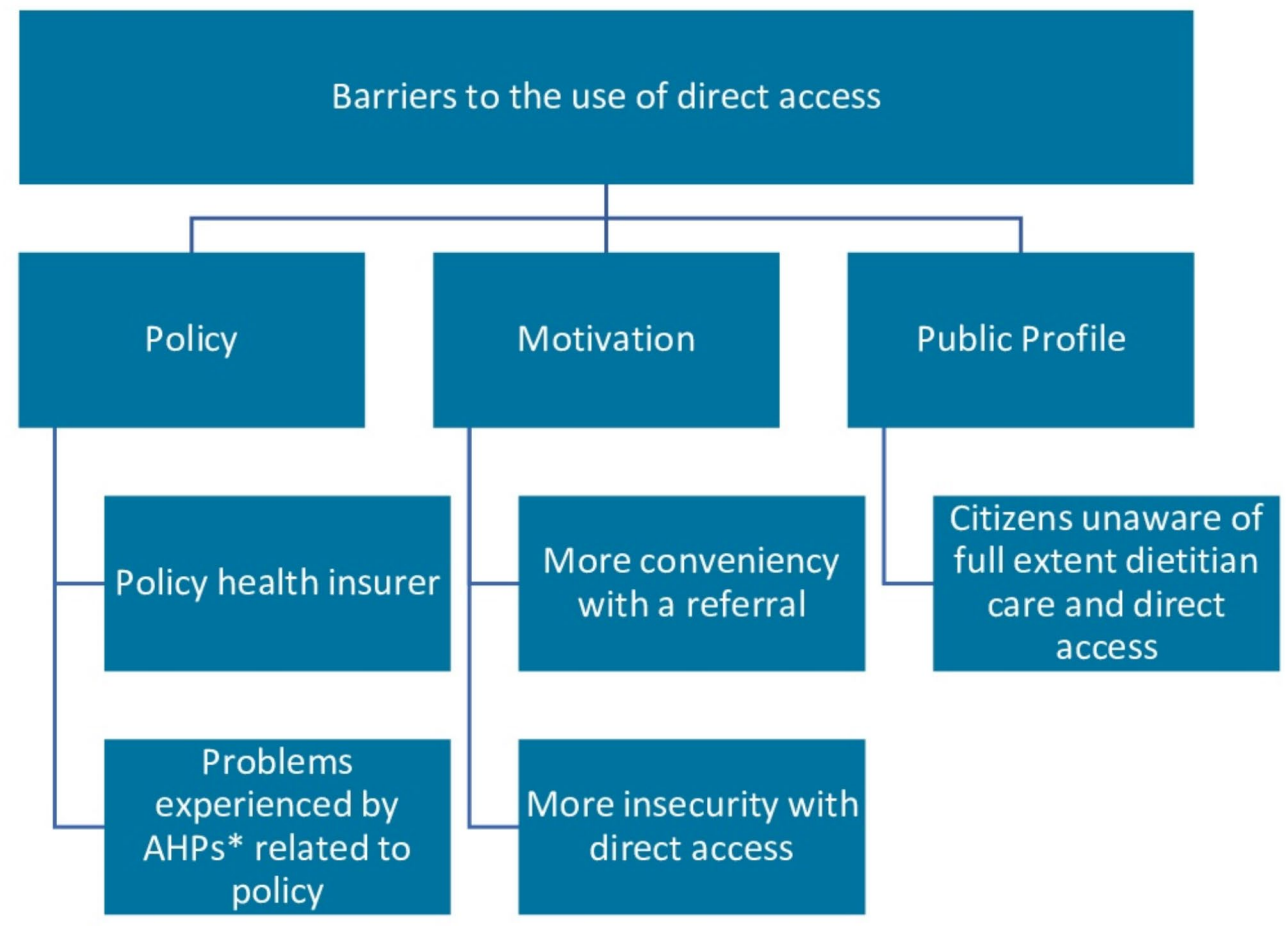
Key themes emerged from the interviews. *AHP allied health professional

In the following section, we will explore the specific barriers both dietitians and physiotherapists experienced with direct access. Key themes and subthemes derived from interviews with allied health professionals and health insurers include **policy**, encompassing policy health insurer and problems experienced by allied health professionals related to policy; **motivation**, covering more conveniency with a referral and more insecurity with direct access; and **public profile**, encompassing citizens unaware of full extend dietitian care and direct access (Fig. 1).

### Policy

The policy theme encompasses rules and agreements that are formalised by health insurers and other agencies.

#### Policy health insurer

Health insurers stated that they impose minimal requirements for the use of direct access. Allied health professionals must undergo specific training to offer these services. This training covers screening, reporting, and the integration of direct access within their practice or organisation. Two insurers noted that a small group of professionals, mainly within speech therapy, dietetics, and occupational therapy, remain untrained. This creates practical challenges, as they must request referrals for all treatments. Additionally, one insurer specified that allied health professionals are not permitted to actively approach patients. For conditions covered by basic health insurance, many insurers still require a referral for physiotherapy, although this is not universally applied. For some health insurers proof of medical indication is necessary, but it can be provided in various forms, such as a printout from the patient’s medical file. With the exception of this fact, health insurers stress that the requirements are the same for all disciplines for the use of direct access.

#### Problems experienced by allied health professionals related to policy

Dietitians pointed out that direct access is often unattractive because it reduces their time available for treatment. Patients are reimbursed for only three hours of dietitian care per year from the basic insurance, while performing the necessary screening for direct access takes at least 15 min from this limited time. With a referral, this screening is not required, leaving an extra 15 min for the actual treatment of the patient. Additionally, most dietitians claim that they are paid less for an intake via direct access compared to an intake via a referral. This is in contrast to physiotherapists, who reported being paid the same or even more for an intake after direct access compared to one with a referral.

Health insurers, however, refute the dietitians claim saying that allied health professionals can claim the same amount for an intake via direct access as for an intake via referral. One health insurer said that allied health providers even receive more for an intake after direct access compared to an intake with a referral:

Quote 1: “*Interviewee: With direct access*,* a screening is included*,* which makes it more comprehensive than a referral. Allied health professionals may not realise they can claim for the screening as well. Direct access is reimbursed at a higher rate.*

*Interviewer: So*,* is it true that an intake through direct access is always reimbursed more than one with a referral?*

*Interviewee: Yes*,* absolutely*.”– Participant 6, health insurer.

Both dietitians and physiotherapists highlighted the cost barrier of direct access for patients. If, after the screening, it turns out that the patient should be treated elsewhere then they will be charged (if they are not insured for physiotherapy or have not reached the limit of their deductible). In addition, they will lose valuable treatment time. By contrast, a visit to the GP is free of charge for patients and does not affect their treatment hours. Allied health professionals suggest that the screening process should be covered by basic insurance, regardless of the outcome of the screening, and not be deducted from the patient’s treatment time.

Another issue raised by dietitians related to policy is that red flags are triggered too easily during screening (Quote 2). This highlights that the mandatory screening list does not always function as intended. When this happens, they must refer the patient to a GP, resulting in lost treatment time. To avoid this, dietitians often advise patients to obtain a referral from the GP in the first place.

*Quote 2: “When I did that training*,* I quickly realised how easily you could spot a red flag. Then I thought*,* it is always my turn. For example*,* any abnormal stool pattern scores a red flag*,* which is almost always the case with intestinal issues. The same applies to polypharmacy. There is a lot of medication being used these days*,* even by relatively healthy people and children. That is another immediate red flag.*”– participant 15, dietitian.

### Motivation

The motivation theme addresses the internal motivation of professionals that influences their decision to use referrals instead of direct access, including convenience and feelings of uncertainty.

#### More convenience with a referral

Dietitians find it more convenient when patients come with a referral from their GP as this already provides necessary background information (like personal data and medical history), saving time otherwise spent gathering this information. This is particularly helpful when patients are unaware of certain aspects of their medical background, which would otherwise require the dietitian to contact the GP. A referral eliminates the need for this extra step:

Quote 3: *“I would like to have that background information. So I request a referral. Although we might also discover it through thorough questioning…”*– Participant 1, dietitian.

Some dietitians noted that relying solely on GP referrals is more convenient from a marketing perspective. Once a relationship is established with a GP who refers a steady stream of patients, there is less need for branding and advertising efforts to attract new clients:

Quote 4: *“I have noticed that referrals provide much greater outreach. Appointments come in consistently*,* significantly expanding your reach. With direct access*,* however*,* you have to put more effort into making sure people know about your services and what you offer*,* which takes time.”*– Participant 11, dietitian.

Another barrier identified by both physiotherapists and dietitians is the inconsistency in policies among health insurers. While policy was discussed in a previous theme, this issue does not pertain to the specific content of an individual insurer’s policies. Instead, it highlights the collective impact of varying policies across all health insurers. This lack of uniformity in these policies creates unnecessary obstacles, making it less convenient and unattractive to use direct access. For example, some insurers restrict certain diagnoses from being treated without a referral, while others allow it. And, according to dietitians, until recently, one insurer did not cover direct access for dietitians at all. These differences between insurers make it challenging to keep track of which insurer covers what. Physiotherapists viewed this lack of uniformity as unnecessary and suggested that removing these complications would make direct access much easier to use (Quote 5). Dietitians saw it as a significant barrier. Some even stopped using or registering patients through direct access altogether, citing repeated denials of claims made through this process (Quote 6). As a result, many now only accept referrals, or register all patients as having been referred, even if they come directly.

Quote 5: *“Each health insurer has its own rules. Some allow direct access for treatments at home*,* while others do not. Over time*,* you get used to it*,* remembering which ones do and which ones do not. However*,* the policy is not uniform*,* while the fees are fairly consistent. Uniform policies would save a lot of time and effort.”*– Participant 17, physiotherapist.

Quote 6: *“Then you submit a claim for direct access*,* and it gets rejected by the insurer. They do not approve it again. So I have decided not to claim anyone through direct access. I register everyone with a referral from a GP or specialist*,* and then it goes through without issues.”*– Participant 12, dietitian.

#### More insecurity with direct access

Both physiotherapists and dietitians noted that a referral provides an added sense of security as the GP has already conducted the initial screening (Quote 7, 8). They both observed that younger professionals often find it challenging to perform a thorough screening on their own. Physiotherapists suggested that pairing a younger therapist with a more experienced one for joint screening, or creating opportunities for the younger therapist to ask questions, could be beneficial in improving their confidence and skills early on.

Quote 7: *“At the GP*,* they are already screened*,* so the GP sends them to us with the idea that we can help. But if they come directly and I have doubts*,* this raises more questions: do you start physiotherapy? Do you send them back to the GP? Do you do both? It brings more uncertainty. Is it something we can actually treat?”–* Participant 10, a young physiotherapist.

Quote 8: *“I can imagine that when you do not yet have much experience*,* you would prefer to have a referral*,* it just gives more clarity and certainty. It can also be partly due to fear*,* like*,* what if I miss something? Then I could be in trouble. So that could also be a reason why some practices prefer referrals*,* out of caution.”*– Participant 3, dietitian.

A dietitian remarked that the perceived differences in the use of direct access between dietitians and physiotherapists can be attributed to the profession itself. She suggested that dietitians experience more insecurity compared to physiotherapists (Quote 9). Two health insurers concurred with this. One insurer noted that physiotherapists are better at interpreting screening results than professionals in other fields. They can better determine whether a referral to a GP is necessary. For instance, speech therapists frequently seek validation from GPs and actively request referrals (Quote 10).

Quote 9: *“Perhaps speech therapists*,* occupational therapists*,* and dietitians tend to lean more toward certainty and may generally be more anxious than physiotherapists. We still want that referral letter. That is something I notice around me as well*.”– Participant 3, dietitian.

Quote 10: “*I have spoken with speech therapists about this*,* and this may partly stem from the nature of their profession…. They often mention that following their screening*,* they encounter findings that prompt them to seek confirmation from a general practitioner. As a result*,* they refer the patient back to the GP to verify the situation and request a referral.*” - Participant 9, health insurer.

Some physiotherapists also emphasised the importance of interpreting screening results. When a red flag appears, it does not necessarily mean the patient must be sent back to the GP. It depends on the context. This is something that comes with experience and needs to be learnt over time:

Quote 11: *“I can also say*,* now that I have been in the profession a bit longer*,* that I often still treat or examine patients after identifying a red flag. I can imagine that a junior colleague might follow the protocol strictly*,* and if the patient answers “yes” to something*,* they immediately think*,* I will refer them back to the GP. I think with more experience as a physiotherapist*,* you can see through this a bit more. You are able to make a better judgement*,* listen to the patient*,* complete the screening*,* finish the anamnesis*,* and conduct a focused physiotherapeutic examination. You get better at this with more experience.”* -Participant 16, physiotherapist.

### Public profile

The public profile theme addresses public awareness of the dietetics profession and the availability of direct access to dietitians, which influences the use of direct access.

#### Citizens unaware of full extent dietitian care and direct access

Dietitians have noted that many individuals, including both patients as well as other HCPs, are unaware of the full extent of their work. The common assumption is that dietitians primarily assist with weight management, which results in patients more frequently being referred by others rather than seeking their services directly. According to dietitians and health insurers, this misconception highlights the need for greater public awareness. This is a responsibility that should be addressed by both the professional association and dietitians themselves. Moreover, many patients remain unaware that dietitians are directly accessible (Quote 12). Dietitians advocating increased awareness propose that health insurers and the government should launch public campaigns to address this issue. However, health insurers disagreed, stating that it is the responsibility of dietitians or GPs to promote this initiative.

Quote 12: “*I think you really need to consider public awareness campaigns by the government or the health insurer. I know that all dietitians say they are directly accessible on their websites and that they all mention it*,* but it is not being read*,* it is not being read at all. Maybe with the new government*,* there should be another awareness campaign: how does healthcare work now?”*– Participant, 15 dietitian.

By contrast to dietitians, most physiotherapists report that patients generally understand their services and know they can access them directly. This awareness is attributed to extensive campaigns conducted when direct access was first introduced, as well as the efficient management of physiotherapy practices (Quote 13, 14). These practices focus heavily on brand awareness and patient recruitment, which some believe makes them more effective than other healthcare disciplines. One health insurer also cited the efficiency of physiotherapy as a reason for its higher rate of the use of direct access compared to other professions (Quote 15).

Quote 13: “*But I think it is also about how that profession positions itself. Physiotherapists were already a bit further along in this*,* which is why they are better at promoting their services than dietitians.”*– Participant 16, physiotherapist.

Quote 14: “*We had a campaign for direct accessibility in physiotherapy*,* with posters. I have never seen anything like that for dietitians or speech therapists*.”– Participant 13, physiotherapist.

Quote 15: “*I have had quite a few discussions over the past few years*,* with various speech therapy practices*,* or received letters*,* and I think… the professional association is*,* indeed*,* focusing a lot on optimising documentation now. However*,* when I compare this to physiotherapy*,* it seems that they were addressing this over ten years ago*,* and it appears that this is only now starting to gain momentum among speech therapists. (…) I believe that in physiotherapy*,* there is a greater effect of market competition*,* which means you must work efficiently and maintain quality and effectiveness*.”– Participant 9, health insurer.

## Discussion

To explore *the barriers to the use of direct access according to dietitians*,* physiotherapists*,* and health insurers*, we conducted interviews with 13 allied health professionals and four health insurers during August and September 2024.

Health insurers stated that they do not impose any specific requirements for the use of direct access and do not oppose its implementation. However, this contrasts with feedback from allied health professionals, who believed that insurers could take steps to simplify its use. The challenge appears not to lie with individual insurers but with the lack of a uniform policy on direct access among health insurers, creating confusion and administrative burden for allied health professionals. As a result, some professionals find it easier to require a referral, which ultimately saves time. Establishing consistent policies across insurers would greatly streamline processes and increase the use of direct access among allied health professionals.

Additionally, there were inconsistencies regarding fees between dietitians and health insurers. Insurers claimed that allied health professionals receive the same or higher payment for an intake following direct access as they do for an intake after a referral. And that this was the same for the different occupations. However, publicly available documents indicate that dietitians receive the same fee for intakes regardless of access type, whereas physiotherapists are reimbursed more for intakes involving direct access screening than for those after a referral [34, 35]. Although this observation is based on data from a single insurer, it highlights a potential disparity between professions. This raises the question of why dietitians are not similarly compensated, given their comparable responsibility in conducting screening.

Dietitians further reported sometimes receiving lower fees for direct access screenings than for intakes via referrals. Although we could not fully verify this discrepancy, one dietitian noted that some insurers reduce payments if a screening identifies a red flag. If accurate, this would contribute to additional ambiguity. While further investigation into this issue is beyond the scope of the current research, we recommend it as an area for future study. Transparent and consistent fee structures are essential, as is appropriate compensation reflecting the additional responsibility, acknowledged by insurers, that is borne by allied health providers. Equal or lower fees for direct access may ultimately discourage its use given the added responsibility it places on providers.

The same issue applies to the screening time being deducted from treatment time. This arrangement does not encourage patients or allied health professionals, particularly dietitians, to use direct access. To make direct access more appealing, it should not come at the expense of treatment time. These findings align with previous literature. A study by an organisational advisory agency investigated the cost, pricing, and affordability of allied health professionals in the Netherlands [17]. In this research, practice owners discussed the potential impact on their practices of limiting direct access. Physiotherapists generally viewed direct access positively, while other allied health professionals rarely used it, preferring referrals instead. The key reasons cited for preferring referrals included limited or no reimbursement for screenings, the unsuitability of screening for children, and the reduction in available treatment time due to screening requirements, particularly for dietetics and occupational therapy.

Another challenge to the use of direct access is the insecurity it can create, particularly for younger professionals. Newly qualified professionals often lack sufficient guidance following their education, which can heighten feelings of uncertainty [36, 37]. One promising suggestion from physiotherapists is to pair younger therapists with more experienced colleagues as a potential solution. However, it remains uncertain whether this approach would be feasible for dietitians, as dietitian practices typically have fewer employees than physiotherapy practice. This would make mentorship more challenging [37]. Addressing this issue may be best achieved through enhanced training in the programme.

Additionally, some dietitians reported challenges with the use of red flags, noting that they are sometimes triggered too easily. Coupled with insecurity and potential difficulties in putting these red flags into context leads to requests for referrals. Revisiting and refining the screening criteria could therefore be a valuable step towards increasing the use of direct access.

Public profile, that is the importance of getting your service known about, is also influencing the use of direct access. According to both physiotherapists and health insurers, physiotherapists are more focused on brand awareness, patient recruitment, improvements in quality, and efficiency than other allied health professionals. This focus has increased public awareness of physiotherapy services and the availability of direct access. By contrast, health insurers, physiotherapists, and dietitians noted that dietitians face challenges in this area. Many patients are unaware of the services dietitians provide or that they can be accessed directly without a referral. Adopting similar management strategies as physiotherapists could help dietitians and other allied health professionals enhance their visibility and encourage the greater use of direct access. However, it is important to acknowledge the inherent differences between these professions. The uptake of direct access in dietetics may never match the levels seen in physiotherapy. Nevertheless, addressing the barriers identified by both physiotherapists and dietitians could increase the use of direct access in both professions.

Direct access appears to increase the workload of some allied health professionals, particularly dietitians, through an unintended consequence of a system designed to alleviate pressure on care. Dietitians report that screening patients under direct access is both time consuming and challenging, reducing the treatment time they can charge for per patient. By addressing the challenges highlighted by allied health professionals, the pressures associated with direct access can be alleviated. This, in turn, could facilitate more frequent use of direct access, ultimately helping to relieve pressure on GP care.

This research highlighted that variations in the use of direct access extend beyond patient demographics, as identified in previous studies [38–40]. The differences among practices and individual allied health professionals also can contribute to these variations. Therefore, policies aimed at increasing direct access should focus on the challenges which allied health professionals have identified.

It is also crucial to note that expanding direct access requires careful consideration, as it may inadvertently increase the risk of overtreatment. Fee-for-service payment models, which often incentivise higher treatment volumes, are a known driver of overuse in healthcare [41]. With easier access to treatment, direct access could encourage higher patient volumes and, potentially, overtreatment if not managed carefully as some symptoms could have a psychosocial origin for example.

### Strengths and limitations

A strength of this study is that we interviewed both health insurers and allied health professionals, two players in healthcare who often attribute issues to each other. We were, thus, able to capture perspectives from both sides. By interviewing these groups alternately, we could bring statements from one group into discussions with the other, providing a richer context. Moreover, to our knowledge, this topic has been underresearched, with limited literature available on the subject.

Initially, we assumed that ‘allied healthcare professionals’ could be considered as a homogeneous group. However, we found no previous research on this topic, meaning we could not verify this assumption in advance. Based on the assumption of a homogeneous group, we expected that a total of 12 participants would likely be sufficient to achieve data saturation, with the option to conduct additional interviews if needed. However, during the analysis, it became evident that dietitians and physiotherapists are distinct professional groups with different experiences and perspectives. Given the high information power of our interviews and time constraints, we proceeded with six/seven participants per group. We believe this sample has strong information power, which is assessed based on the following criteria: The aim of the study; its sample specificity; the use of established theory; the quality of dialogue; and the analysis strategy [42]. Our study has a narrow, focused aim, which enhances the relevance of the information. Moreover, the allied health professionals and health insurers involved possess extensive experience and knowledge on the topic, resulting in a high degree of sample specificity. Furthermore, our interview guide was informed by the CFIR model, which provided a solid theoretical foundation and, we employed thematic analyses to examine the results. Therefore, despite a smaller sample size per group, the information power remains high.

Although, this study is exploratory rather than exhaustive, we consider it unlikely that the absence of additional interviews to achieve saturation affected the results. Moreover, while dietitians and physiotherapists expressed different perspectives on direct access, both groups largely identified similar challenges and disadvantages associated with direct access, as reflected in the derived themes.

The non-random sampling approach most likely resulted in some overrepresentation of individuals with a strong interest in, or strong opinions about, direct access, whether supportive or opposed. Moreover, the study may have amplified the polarisation of opinions between physiotherapists and dietitians. Physiotherapists, who are generally more supportive of direct access, may have been disproportionately represented by participants with strongly positive attitudes. Conversely, dietitians, who tend to be less favourable, may have been overrepresented by those with strongly negative attitudes. This imbalance could distort the perception of a professional consensus and exaggerate the differences in opinion between these groups.

In addition, three of the health insurer delegates had only five or fewer years of experience within their organisations, raising questions about the depth of their understanding of the overall operations of the health insurer. However, we specifically invited delegates with knowledge of the insurer’s procurement policies, and policies related to direct access. Therefore, it is reasonable to assume that these delegates can adequately represent the health insurer in this context.

### Future research

Initially, we considered conducting focus groups to allow direct interaction between health insurers and allied health professionals. However, interviews proved to be the right choice, as some allied health professionals indicated they might not have shared certain information if speaking directly to a health insurer. However, future research should include focus groups with GPs and allied health professionals to clarify referral practices. Studies based on different scenarios could also explore cases where patients are referred unnecessarily according to the GP. This could help examine factors such as uncertainty.

Additionally, we recommend conducting larger-scale studies on the use of direct access and perceived barriers by these and other allied health professionals, such as through questionnaires, now that we have a clearer understanding of the challenges faced by them.

Another valuable area of study would be to compare, across different occupations, less and more experienced allied health professionals in order to assess differences in their screening skills. Additionally, it would be insightful to examine how much emphasis educational programmes place on developing these skills and to explore potential areas for improvement in training.

Finally, incorporating the patient’s perspective on this topic would add valuable depth to the study. Questions such as what motivates patients to use direct access, how frequently they seek care without a formal referral but upon the advice of a GP, and how they perceive being referred back to the GP due to a red flag, would provide important insights.

## Conclusion

Allied health professionals face several challenges in using direct access. These challenges are related to policy, motivation, and one’s public profile. The barriers included limited problems with reimbursement for screenings and reduced treatment time due to screening obligations. While health insurers stated that they do not impose specific requirements or oppose the implementation of direct access, differences in perspectives between insurers and allied health professionals remain. Additionally, uncertainty about screening skills posed a significant challenge for allied health professionals. Moreover, requesting a referral is sometimes more convenient than using direct access, as it involves fewer regulations and saves time for allied health professionals. A further issue is the need for an improved public profile in order to enhance patient awareness of direct access. Notably, direct access has inadvertently increased the workload for some allied health professionals, particularly dietitians—an unintended outcome of a system intended to reduce the pressure on care.

Addressing these challenges and bridging the gap between the views of health insurers and allied health professionals are crucial for making the best use of direct access. These efforts could help reduce the pressure on primary care and improve the efficiency of healthcare delivery.

## Electronic supplementary material

Below is the link to the electronic supplementary material.

Supplementary Material 1: Appendix A– Interview guide allied health professionals

Supplementary Material 2: Appendix B– Interview guide health insurers

Supplementary Material 3: Appendix C– Codebook allied health professionals

Supplementary Material 4: Appendix D– Codebook health insurers

## Data Availability

The qualitative data collected and analysed during the current study concerns individual interview reports with allied health professionals and health insurers which fall under the GDPR and are therefore not publicly available. However, they are available on reasonable request from rvb@nivel.nl, the executive board of Nivel, under the name “ALG-017 Right Care in the Right Place”.
