# Supplementary material for: Barriers to the use of direct access according to allied health professionals; an exploration among Dutch physiotherapists, dietitians, and health insurers
Source: BMC Prim Care. 2025 Apr 25;26:127. doi: 10.1186/s12875-025-02816-y (PMC12032724; doi:10.1186/s12875-025-02816-y)
Supplement: Supplementary file 1 — Supplementary Material 1: Appendix A– Interview guide allied health professionals [file 12875_2025_2816_MOESM1_ESM.docx]

**Appendix A – Interview guide allied health professionals***Introduction*

- Could you introduce yourself?
- Could you tell me a bit about the practice where you work? (e.g., number of therapists, patient population, health centre setting?)

*General*

- How do most patients typically enter the practice (via direct access or through referrals)?
- What differences do you notice when a patient comes in through direct access versus a referral?
- What do you see as the advantages and disadvantages of direct access compared to referrals?

*Therapist/Practice Perspective*

- How do you perceive direct access? Why? *Do you have a preference for direct access or a referral?*
- What is the policy within your practice regarding direct access? Who or what determines this policy?
- Is direct access used widely in your practice? Why or why not?
- To what extent do you believe there are differences between practices in the use of direct access? What factors might explain these differences?

*If no differences: Our previous research suggests significant differences between practices in the use of direct access. What might explain these differences, in your view?*

- For dietitians: In our dataset, around 30% of practices never used direct access, with all patients coming via referral. What might explain this?

*External Factors Affecting the use of direct access*

*Health Insurer/Government Policies*

- In what ways could the government or health insurers make the use of direct access more appealing?

*Do you find health insurers to be a limiting factor in the use of direct access?*

*How much impact do government or insurer policies have on the use of direct access?*

- Have you experienced a health insurer rejecting direct access? Could you provide examples?
- To what extent do administrative burdens hinder the use of direct access?

*How much more/less time do you spend on a patient coming in via direct access compared to a patient coming in via referral?*

*Patient-Related Factors*

- Which types of patients enter through direct access, and which do not?
- What reasons do patients have for choosing or not choosing direct access?
- How aware do you believe patients are of direct access?
- Should more patients be informed about direct access? *Why?*
  *If yes: How should this awareness be raised?*
- Do you inform patients about direct access?

*Disciplinary Differences*

- *Previous research shows that around 70% of physiotherapy patients come through direct access, whereas about 70% of dietetics and speech therapy patients come via referral.* Why do you think direct access is more prevalent in physiotherapy?

*Promoting direct access*

- Should direct access use be promoted in your opinion? *Why or why not? Is there a professional need or benefit in terms of recognition?*
  If yes:
- How could direct access usage be promoted?
- What is needed to achieve this?
- Who should be involved in this effort?
