# Supplementary material for: Barriers to the use of direct access according to allied health professionals; an exploration among Dutch physiotherapists, dietitians, and health insurers
Source: BMC Prim Care. 2025 Apr 25;26:127. doi: 10.1186/s12875-025-02816-y (PMC12032724; doi:10.1186/s12875-025-02816-y)
Supplement: Supplementary file 2 — Supplementary Material 2: Appendix B– Interview guide health insurers [file 12875_2025_2816_MOESM2_ESM.docx]

**Appendix B – Interview guide health insurers**
*Introduction*

- Would you like to introduce yourself? Could you tell us about your job title and your connection to the topic we are discussing today?

*General*

- What agreements exist in allied health professionals contracts regarding direct access?
- Are these agreements consistent for all allied health professionals?
- Is there a difference in direct access for healthcare providers with and without contracts?
- What differences do health insurers notice when a patient enters through direct access compared to a referral?
- Can all patients access direct access with allied health professionals? Why or why not?
- Are there restrictions on direct access use for patients or providers? Does this apply to all disciplines?
- To what extent are claims reviewed for direct access, and could direct access claims be rejected? Are there differences between disciplines? What are these based on?

*Reasons for Facilitating direct access*

- What are the motivations for the health insurer to facilitate direct access?

*Benefits for the health insurer, therapists, or patients; Quality of care; Financial/resource considerations.
Why has* direct access *not always been facilitated?*

- What barriers exist in facilitating direct access?
  *Laws/regulations; Attitudes of paramedics or general practitioners: How do they view direct access?; Quality; Costs: are costs different for direct access versus referral? Who bears these costs (e.g., society, health insurer, patient)? ; Patients: To what extent do patients use direct access, and are they aware of it? Should they be better informed? Who is responsible for this?; Practicality: Feasibility in practice and administrative burden?*

*Differences between practices and occupations*

*Our previous research showed that 70% of physiotherapy patients came through direct access, unlike dietetics and speech therapy, where about 70% were referred.*

- Is this pattern familiar to you?
- How can differences between disciplines in the use of direct access be explained?

*Does direct access function similarly across disciplines? Do all disciplines meet the same conditions or requirements (e.g., administrative load, reimbursement for direct access)?*

- How can differences in the use of direct access between practices be explained?

*Promoting direct access*

- Promoting direct access could reduce unnecessary GP visits, leading to more efficient patient flows in primary care. Do you agree?
- Should the use of direct access be encouraged? Why or why not?

*Could direct access use be promoted for all disciplines? Why or why not?*If yes:

- How could direct access be promoted, and what would be needed?
- Who would be involved in this? What role would the health insurer play?
