# Supplementary material for: Barriers to the use of direct access according to allied health professionals; an exploration among Dutch physiotherapists, dietitians, and health insurers
Source: BMC Prim Care. 2025 Apr 25;26:127. doi: 10.1186/s12875-025-02816-y (PMC12032724; doi:10.1186/s12875-025-02816-y)
Supplement: Supplementary file 3 — Supplementary Material 3: Appendix C– Codebook allied health professionals [file 12875_2025_2816_MOESM3_ESM.docx]

**Appendix C – Codebook allied health professionals**

1. Referral or direct access
2. Target group
   - Direct access
   - Referrals
3. Working method
   - Direct access
   - Referrals
   - No difference
4. Problems with direct access
   - System
   - Other HCPs
   - Allied health professional
   - Health insurer
   - Patient
5. Advantages direct access
   - Health insurer
   - GP
   - Allied health professional
   - Patient
   - Society
6. Opinion/preference referral/direct access
7. Explanations differences between practices
8. Explanations differences between disciplines
9. Promoting direct access
   - How
   - Who
   - Why
