# Supplementary material for: Barriers to the use of direct access according to allied health professionals; an exploration among Dutch physiotherapists, dietitians, and health insurers
Source: BMC Prim Care. 2025 Apr 25;26:127. doi: 10.1186/s12875-025-02816-y (PMC12032724; doi:10.1186/s12875-025-02816-y)
Supplement: Supplementary file 4 — Supplementary Material 4: Appendix D– Codebook health insurers [file 12875_2025_2816_MOESM4_ESM.docx]

**Appendix D – Codebook health insurers**

1. Policy regarding direct access.
2. Why facilitating direct access
3. Differences between occupations
   - Factual differences
   - Thoughts about differences
4. Checking what was said by allied health professionals
   - Amount direct access
   - Time for screening
   - Rejecting direct access
5. Promoting direct access
   - Why
   - How
   - Who
6. Problems with direct access
7. Explanations differences between practices
